# LncRNA H19 inhibits autophagy by epigenetically silencing of DIRAS3 in diabetic cardiomyopathy

**DOI:** 10.18632/oncotarget.13637

**Published:** 2016-11-26

**Authors:** Chuanjun Zhuo, Ronghuan Jiang, Xiaodong Lin, Mingjing Shao

**Affiliations:** ^1^ Department of Psychological Medicine, Wenzhou Seventh People's Hospital, Wenzhou, China; ^2^ Institute of Mental Health, Jining Medical University, Jining, China; ^3^ Department of Psychological Medicine, Tianjin Anding Hospital, Tianjin, China; ^4^ Department of Psychological Medicine, Tianjin Anning Hospital, Tianjin, China; ^5^ Department of Psychological Medicine, Chinese People's Liberation Army (PLA) General Hospital, Chinese PLA (People's Liberation Army) Medical School, Beijing, China; ^6^ Department of Cardiology, China-Japan Friendship Hospital, Beijing, China

**Keywords:** H19, DIRAS3, autophagy, diabetic cardiomyopathy

## Abstract

We previously generated a rat model of diabetic cardiomyopathy and found that the expression of long non-coding RNA H19 was downregulated. The present study was aimed to explore the pathogenic role of H19 in the development of diabetic cardiomyopathy. Overexpression of H19 in diabetic rats attenuated cardiomyocyte autophagy and improved left ventricular function. High glucose was found to reduce H19 expression and increase autophagy in cultured neonatal cardiomyocytes. The results of RNA-binding protein immunoprecipitation showed that H19 could directly bind with EZH2 in cardiomyocytes. The chromatin immunoprecipitation assays indicated that H19 knockdown could reduce EZH2 occupancy and H3K27me3 binding in the promoter of DIRAS3. In addition, overexpression of H19 was found to downregulate DIRAS3 expression, promote mTOR phosphorylation and inhibit autophagy activation in cardiomyocytes exposed to high glucose. Furthermore, we also found that high glucose increased DIRAS3 expression in cardiomyocytes and DIRAS3 induced autophagy by inhibiting mTOR signaling. In conclusion, our study suggested that H19 could inhibit autophagy in cardiomyocytes by epigenetically silencing of DIRAS3, which might provide novel insights into understanding the molecular mechanisms of diabetic cardiomyopathy.

## INTRODUCTION

Diabetes is a common metabolic disorder which is characterized by hyperglycemia and deficient secretion or action of endogenous insulin. Prospective population-based studies have shown that the risk of heart failure is increased significantly by diabetes [1, 2]. Diabetic cardiomyopathy (DCM) is one of the important cardiovascular complications of diabetes and is defined as myocardial dysfunction occurring in diabetic patients without coronary artery disease and hypertension [3]. In recent years, it has been reported that patients with schizophrenia are prone to metabolic syndrome and diabetic cardiovascular diseases. Although a variety of morphological characteristics have been identified associated with DCM, the underlying molecular mechanisms of DCM are still not fully understood.

Long non-coding RNAs (lncRNAs) are defined as transcribed RNA molecules which are longer than 200 nucleotides, but they have no protein-coding function [4]. LncRNAs can regulate gene expression via several different mechanisms. First, they can directly act on the genomic DNA to regulate expression. Second, they can interact with proteins, namely transcription factors and some RNA-binding proteins, to indirectly regulate transcription. Third, they can act as competing endogenous RNAs to sponge miRNAs and regulate the derepression of miRNA targets [5]. In the present study, we generated a rat model of DCM and found that H19 expression was remarkably downregulated. The H19 is a maternally expressed imprinted gene and plays critical roles in the embryonal development and growth control [6]. This study was designed to explore the pathogenic role of H19 in the development of DCM.

## RESULTS

### H19 is involved in the suppression of cardiomyocyte autophagy

**Figure 1:**
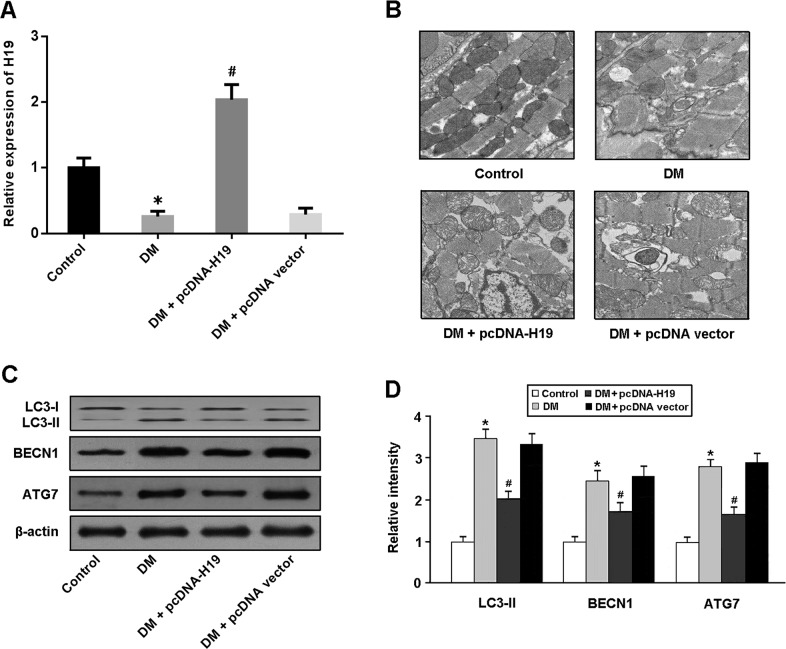
H19 is involved in the suppression of cardiomyocyte autophagy in diabetic rats **A.** The mRNA expression of H19 in cardiac tissue was detected by real-time PCR. **B.** Cardiomyocyte autophagy was assessed by observation of autophagosomes using transmission electron microscopy. **C, D.** The expression of autophagy-related protein (LC3, BECN1 and ATG7) was detected by Western blot. ^*^ P<0.05 compared with control; ^#^ P<0.05 compared with DM (n=5 rats per group).

The expression of H19 was markedly downregulated in the myocardium of diabetic rats and upregulated following injection with lentivirus pcDNA-H19 (Figure 1A). Cardiomyocyte autophagy was assessed by observation of autophagosomes using TEM and detection of autophagy-related protein expression. Our results showed that autophagy was significantly activated in response to hyperglycemia, and enforced expression of H19 in diabetic rats decreased the number of autophagosomes and reduced the expression of LC3-II, BECN1 and ATG7 (Figure 1B–1D).

### H19 overexpression improves cardiac function in diabetic rats

**Figure 2:**
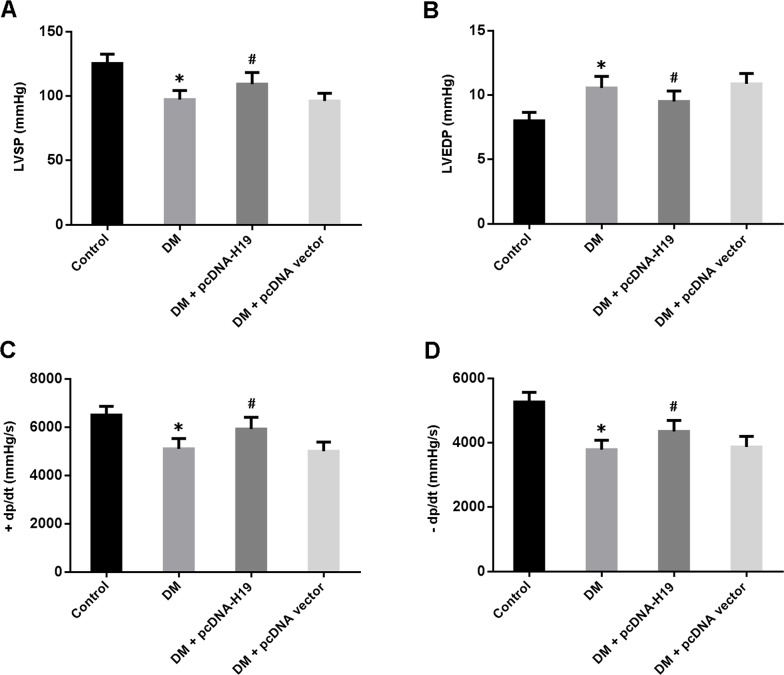
Overexpression of H19 improves cardiac function in diabetic rats **A.** left ventricular systolic pressure (LVSP); **B.** left ventricular end-diastolic pressure (LVEDP); **C.** maximal ascending rate of left ventricular pressure (+dp/dt); **D.** maximal descending rate of left ventricular pressure (-dp/dt). ^*^ P<0.05 compared with control; ^#^ P<0.05 compared with DM (n=5 rats per group).

Cardiac systolic and diastolic function was evaluated by hemodynamic measurements and the data are presented in Figure 2. Our findings suggested that LVSP and ±dp/dt were reduced and LVEDP was elevated in diabetic rats, while enforced expression of H19 could significantly improve left ventricular dysfunction associated with hyperglycemia.

### H19 is involved in high glucose-induced autophagy by regulating DIRAS3

**Figure 3:**
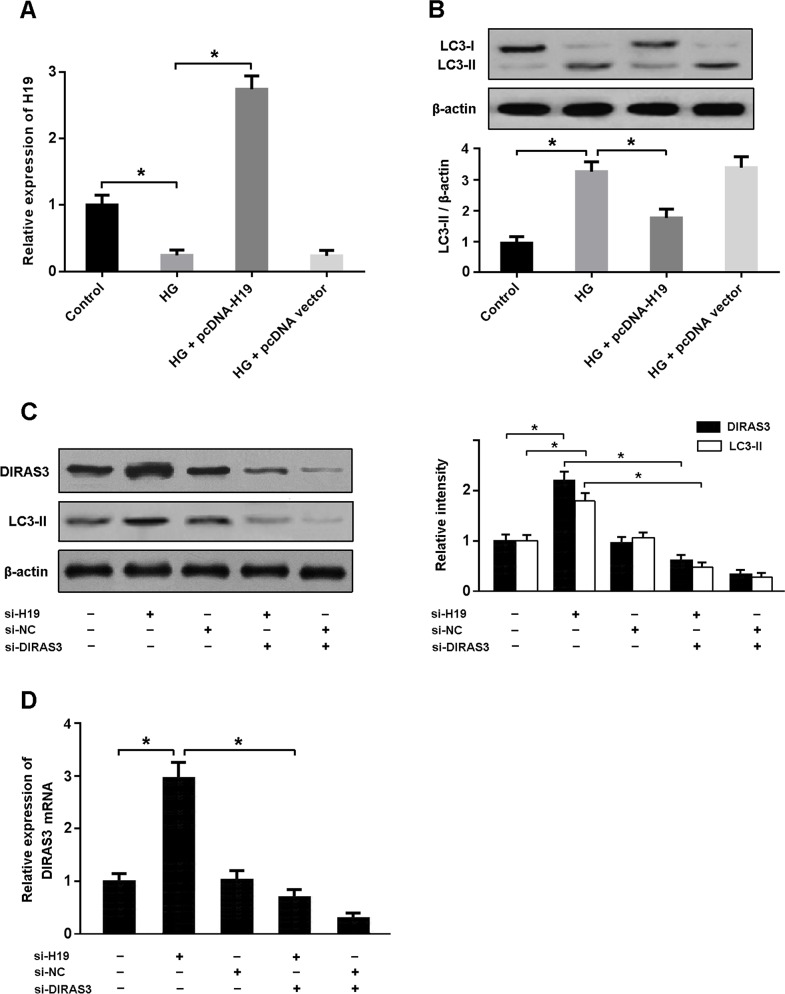
H19 is involved in high glucose-induced autophagy by regulating DIRAS3 **A.** Cardiomyocytes were transfected with adenoviral pcDNA-H19 or empty vector and then exposed to high glucose (HG, 30 mmol/L) for 48 hours. The H19 expression was determined by real-time PCR. **B.** Western blot analysis of LC3 as an important marker of autophagy. **C, D.** Cardiomyocytes were infected with adenoviral H19 siRNA or control and then transfected with adenoviral DIRAS3 siRNA. The expression of DIRAS3 and LC3-II was detected by real-time PCR and Western blot. ^*^ P<0.05 (n = 5 independent experiments).

High glucose was found to be associated with decreased H19 expression and activated cardiomyocyte autophagy (Figure 3A, 3B). The mRNA and protein expression of DIRAS3 was upregulated in cardiomyocytes transfected with H19 siRNA, consequently leading to increased autophagy. However, knockdown of DIRAS3 could inhibit autophagy in cardiomyocytes with H19 siRNA transfection, suggesting that H19 downregulation induces autophagy by enhancing the expression of DIRAS3 (Figure 3C, 3D).

### DIRAS3 is epigenetically silenced by H19 in cardiomyocytes

**Figure 4:**
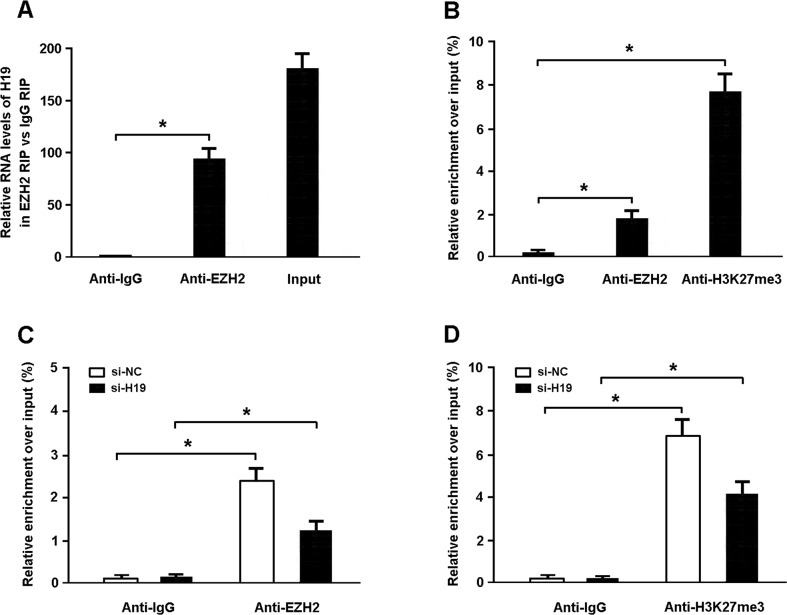
DIRAS3 is epigenetically silenced by H19 in cardiomyocytes **A.** The RIP assay was performed to verify whether H19 could directly bind with EZH2 in cardiomyocytes. **B.** The ChIP assay was conducted to confirm whether EZH2 could directly bind to DIRAS3 promoter region and mediate H3K27me3 modification in cardiomyocytes. **C, D.** ChIP-qPCR of EZH2 occupancy and H3K27me3 binding in the promoter of DIRAS3 in cardiomyocytes transfected with H19 siRNA or scrambled siRNA. ^*^ P<0.05 (n = 5 independent experiments).

To further investigate whether H19 represses DIRAS3 expression through binding to PRC2, we performed RIP assays and found that H19 could directly bind with EZH2 in cardiomyocytes (Figure 4A). In addition, the results of ChIP assays showed that EZH2 could directly bind to DIRAS3 promoter region and mediate H3K27me3 modification (Figure 4B). Furthermore, knockdown of H19 could reduce EZH2 occupancy and H3K27me3 binding in the promoter of DIRAS3 in cardiomyocytes (Figure 4C, 4D).

### High glucose promotes autophagy by regulating H19/DIRAS3 pathway

**Figure 5:**
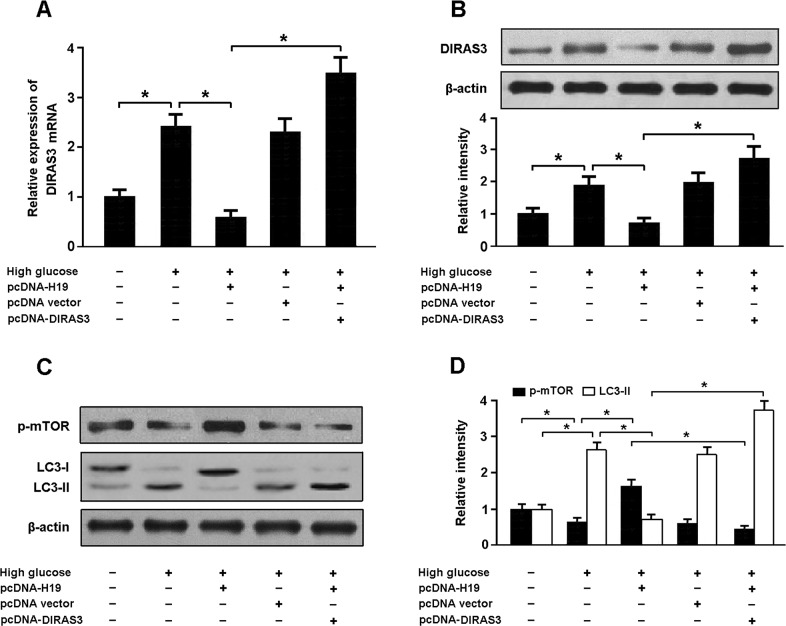
High glucose promotes autophagy by regulating H19/DIRAS3 pathway **A, B.** Cardiomyocytes were transfected with pcDNA-H19 and/or pcDNA-DIRAS3 prior to exposure to high glucose and the expression of DIRAS3 was analyzed using real-time PCR and Western blot. **C, D.** Western blot analysis of phosphorylated mTOR (Ser2448) and LC3-II in cardiomyocytes with different treatments. ^*^ P<0.05 (n = 5 independent experiments).

Cardiomyocytes were transfected with pcDNA-H19 and/or pcDNA-DIRAS3 prior to exposure to high glucose. Our results indicated that high glucose increased DIRAS3 expression and cardiomyocyte autophagy and reduced mTOR phosphorylation. Overexpression of H19 downregulated DIRAS3 expression, promoted mTOR phosphorylation and suppressed autophagy activation in cardiomyocytes exposed to high glucose. Moreover, enforced expression of H19 and DIRAS3 could induce autophagy in cardiomyocytes by inhibiting mTOR signaling (Figure 5A–5D).

## DISCUSSION

In this study, we generated a streptozocin-induced diabetic rat model to explore the potential role of lncRNA H19 in the pathogenesis of DCM. Our results indicated that H19 was significantly downregulated in the myocardium of diabetic rats, which might be associated with increased cardiomyocyte autophagy and impaired cardiac function. We then further explored the molecular mechanisms by which H19 participates in the modulation of high glucose-induced autophagy using cultured neonatal cardiomyocytes. Our findings revealed that H19 could inhibit cardiomyocyte autophagy by epigenetically silencing of DIRAS3.

H19 is a maternally expressed lncRNA and plays critical roles in cellular proliferation, apoptosis, differentiation and invasion. H19 regulates bladder cancer metastasis through its association with EZH2 [7]. This association leads to the activation of Wnt/β-catenin and the downregulation of E-cadherin. In addition, H19 can act as an endogenous competing RNA to sequester miR-106a and mir-let7 family [8, 9]. Furthermore, H19 can serve as a precursor of miR-675 and regulate carcinogenesis, progression and metastasis of several types of cancers [10–12].

In recent years, PRC2 has been found to be involved in various biological processes, including proliferation, apoptosis and differentiation [13]. EZH2 is a key catalytic subunit of PRC2 and acts as a histone methyltransferase inducing histone H3 lysine 27 trimethylation (H3K27me3) to target genes [14]. As a significant epigenetic regulator, EZH2 is highly expressed in a wide range of human cancers and mediates the expression of target genes responsible for cell cycle progression, proliferation, differentiation, and neoplastic cell transformation [15]. In this study, our results indicated that H19 could epigenetically repress DIRAS3 transcription in cardiomyocytes by binding with PRC2 and recruiting it to DIRAS3 promoter region.

Autophagy is a highly regulated catabolic process in which long-lived proteins and dysfunctional organelles are sequestered into lysosomes and degraded [16]. It has been well documented that autophagy could be activated by AMPK signaling and suppressed by mTOR signaling [17, 18]. In recent years, accumulating evidence has revealed that autophagy plays crucial roles in the pathogenesis of DCM [19, 20]. DIRAS3, also termed ARHI, is widely expressed in human epithelial cells from different organs. There is growing evidence that DIRAS3 is involved in the activation of autophagy. DIRAS3 is involved in the formation of autophagic vesicles by increasing ATG4 expression and colocalizing with LC3-II in autophagic vesicle membranes [21]. In addition, DIRAS3 also alters intracellular signaling pathways PI3K/AKT/mTOR and AMPK/TSC1/TSC2, which are involved in the modulation of autophagy [21]. A recent study showed that under nutrient-poor conditions, DIRAS3 expression is upregulated, which subsequently displaces Bcl-2 from BECN1, forms DIRAS3-BECN1 heterodimers and promotes interaction of BECN1 monomers with PIK3C3 and ATG14, thereby increasing PIK3C3 kinase activity and ultimately inducing autophagy [22]. In this study, our results revealed that DIRAS3 could induce autophagy in cardiomyocytes exposed to high glucose by inhibiting mTOR signalling.

In summary, our study demonstrates that DIRAS3 is a novel target of repression by EZH2-mediated H3K27me3 and is epigenetically silenced by H19 in cardiomyocytes, which will provide new insights into understanding the molecular mechanisms of DCM.

## MATERIALS AND METHODS

### Animal model and treatment

All experiments were approved by the Animal Ethics Committee and were carried out in accordance with the Guide for the Care and Use of Laboratory Animals. Male Sprague–Dawley rats weighing 200–250g were obtained and diabetic rat model was induced by a single intraperitoneal injection of streptozotocin (60 mg/kg) as previously described [23]. The tail vein blood glucose levels were detected using a glucometer. Rats with blood glucose levels ≥16.7 mmol/l were in accordance with the diagnostic criteria for diabetes. Diabetic rats were intracoronary injected with lentivirus pcDNA-H19 (DM+pcDNA-H19 group) or empty vector (DM+pcDNA-vector group). After 3 months of feeding, the various experiments were performed.

### Cardiomyocyte culture

Neonatal ventricular myocytes were isolated from 1-2 days old rats as previously described [24]. Briefly, myocardial tissues were surgically removed and dispersed in a series of incubations at 37°C in D-Hanks buffered solution containing 1.2 mg/mL pancreatin and 0.14 mg/mL collagenase (GIBCO, USA). After centrifugation, the cells were suspended in Dulbecco's modified Eagle medium/F-12 containing 10% heat-inactivated foetal bovine serum, 100 U/mL penicillin, 100 mg/mL streptomycin, and 0.1 mM bromodeoxyuridine. The dissociated cells were pre-plated at 37°C for 1 h to separate cardiomyocytes by adherence of cardiac fibroblasts. The cardiomyocytes were collected and diluted to 1×10^6^ cells/mL and plated in 1% gelatin-coated different culture dishes. Neonatal cardiomyocytes were incubated at 37°C and 5% CO_2_ in a humidified chamber. In the following experiments, cardiomyocytes were exposed to high glucose (30 mM) for the mechanism exploration.

### Hemodynamic study

Hemodynamic measurements were carried out after 12 weeks of diabetes induction. Rats were anesthetized by intraperitoneal injection of pentobarbital, and a 2F catheter connected with a polygraph system was introduced into the left ventricle via the right carotid artery. The following measurements were obtained: left ventricular systolic pressure (LVSP), left ventricular end-diastolic pressure (LVEDP), maximal ascending rate of left ventricular pressure (+dp/dt), and maximal descending rate of left ventricular pressure (-dp/dt).

### Transmission electron microscopy (TEM)

Rat myocardial tissues were cut into small pieces, and then fixed in 2.5% glutaraldehyde, post-fixed in 1% osmium tetroxide, dehydrated in an ascending series of alcohols, and embedded in epoxy resin. The ultrathin sections were cut, stained with uranyl acetate and lead citrate, and examined under a Philips CM120 transmission electron microscope.

### Chromatin immunoprecipitation (ChIP)

The ChIP assay was carried out using the EZ-ChIP Kit (Millipore, USA) following the manufacturer's protocol. Briefly, cardiomyocytes were treated with formaldehyde and incubated for 10 minutes to generate DNA-protein cross-links. Cell lysates were then sonicated to generate chromatin fragments of 200-300 bp and immunoprecipitated with EZH2 and H3K27me3-specific antibodies (Millipore, USA) or negative control IgG (Millipore, USA). The precipitated chromatin DNA was purified and subjected to quantitative PCR analysis for enrichment of the target sequences.

### RNA-binding protein immunoprecipitation (RIP)

Cardiomyocytes were lysed in RIP lysis buffer, following incubation with RIP buffer containing magnetic beads conjugated with anti-EZH2 antibody (Millipore, USA) or negative control IgG. Anti-SNRNP70 (Millipore, USA) was used as positive control for the RIP procedure. The samples were incubated with Proteinase K with shaking to digest the protein and then immunoprecipitated RNA was isolated. The RNA concentration was measured using a NanoDrop (Thermo Scientific) and the RNA quality assessed using a bioanalyser. Furthermore, purified RNA was subjected to real-time PCR to determine the presence of binding targets using respective primers.

### Real-time PCR

Total RNA was extracted from heart tissues and neonatal cardiomyocytes using TRIzol Reagent (Invitrogen, USA), cDNA was synthesized using SuperRT One Step RT–PCR Kit (Invitrogen, USA) and subjected to real-time PCR using SYBR Green PCR Master Mix (TOYOBO, Japan) with 7300 Fast Real-Time PCR System (Applied Biosystems, USA), and GAPDH was used as a reference gene. The following primers were used: GAPDH, 5′-TGCCCAGAACATCATCCCT-3′ and 5′-GGTCCTCA GTGTAGCCCAAG-3′; H19, 5′-TATCGGACTCCAGAG GGATT-3′ and 5′-GGCATACAGTGCACCAAGTC-3′; DIRAS3, 5′-CGGCTGTGCTACGAGAAGA-3′ and 5′-AAACTTAGACGGGCAGGTGA-3′. Real-time PCR was performed in triplicate, and the relative expression of genes was calculated using the 2^−ΔΔCT^ method.

### Western blotting

The samples were homogenized in 0.1% SDS buffer containing 125 mM NaCl, 10 mM EDTA, 25 mM HEPES, 10 mM Na_3_VO_4_, 0.5% deoxycholic acid, 0.1% SDS, 1% Triton X-100 with Complete™ protease inhibitor cocktail (Roche, USA). The lysate was centrifuged at 12,000 rpm for 15 min. The supernatant was then collected and the protein concentration was determined using protein assay kit (Bio-Rad, USA). The extracted protein was separated on SDS-PAGE gel, and transferred onto PVDF membrane (Millipore, USA). The membrane was blocked with 5% bovine serum albumin for 1 h to reduce non-specific binding. Then, the blot was incubated with the primary antibody for 12 h at 4°C. The antibodies were purchased from Cell Signaling Technology and were used at manufacturer-recommended dilutions. After washing, the blot was incubated with HRP-conjugated secondary antibody (Santa Cruz, USA) for 1 h at room temperature. Finally, the signal was detected using the enhanced chemiluminescence kit (Amersham Biosciences) and exposed to X-film.

### Statistical analysis

In this study, data are expressed as mean ± SD. All statistical analyses were performed using SPSS software. Statistical differences between two groups were determined by Student's t-test, and statistical differences among more than two groups were determined by Analysis of Variance (ANOVA) followed by SNK-q method. *P* <0.05 was considered statistically significant.

## References

[1] Bahtiyar G, Gutterman D, Lebovitz H (2016). Heart Failure: a Major Cardiovascular Complication of Diabetes Mellitus. Curr Diab Rep.

[2] From AM, Leibson CL, Bursi F, Redfield MM, Weston SA, Jacobsen SJ, Rodeheffer RJ, Roger VL (2006). Diabetes in heart failure: prevalence and impact on outcome in the population. Am J Med.

[3] Falcão-Pires I, Leite-Moreira AF (2012). Diabetic cardiomyopathy: understanding the molecular and cellular basis to progress in diagnosis and treatment. Heart Fail Rev.

[4] Mercer TR, Dinger ME, Mattick JS (2009). Long non-coding RNAs: insights into functions. Nat Rev Genet.

[5] Bak RO, Mikkelsen JG (2014). miRNA sponges: soaking up miRNAs for regulation of gene expression. Wiley Interdiscip Rev RNA.

[6] Gabory A, Jammes H, Dandolo L (2010). The H19 locus: role of an imprinted non-coding RNA in growth and development. Bioessays.

[7] Luo M, Li Z, Wang W, Zeng Y, Liu Z, Qiu J (2013). Long non-coding RNA H19 increases bladder cancer metastasis by associating with EZH2 and inhibiting E-cadherin expression. Cancer Lett.

[8] Imig J, Brunschweiger A, Brümmer A, Guennewig B, Mittal N, Kishore S, Tsikrika P, Gerber AP, Zavolan M, Hall J (2015). miR-CLIP capture of a miRNA targetome uncovers a lincRNA H19-miR-106a interaction. Nat Chem Biol.

[9] Kallen AN, Zhou XB, Xu J, Qiao C, Ma J, Yan L, Lu L, Liu C, Yi JS, Zhang H, Min W, Bennett AM, Gregory RI, Ding Y, Huang Y (2013). The imprinted H19 lncRNA antagonizes let-7 microRNAs. Mol Cell.

[10] Keniry A, Oxley D, Monnier P, Kyba M, Dandolo L, Smits G, Reik W (2012). The H19 lincRNA is a developmental reservoir of miR-675 that suppresses growth and Igf1r. Nat Cell Biol.

[11] Gao WL, Liu M, Yang Y, Yang H, Liao Q, Bai Y, Li YX, Li D, Peng C, Wang YL (2012). The imprinted H19 gene regulates human placental trophoblast cell proliferation via encoding miR-675 that targets Nodal Modulator 1 (NOMO1). RNA Biol.

[12] Dey BK, Pfeifer K, Dutta A (2014). The H19 long noncoding RNA gives rise to microRNAs miR-675-3p and miR-675-5p to promote skeletal muscle differentiation and regeneration. Genes Dev.

[13] Margueron R, Reinberg D (2011). The Polycomb complex PRC2 and its mark in life. Nature.

[14] Cao R, Wang L, Wang H, Xia L, Erdjument-Bromage H, Tempst P, Jones RS, Zhang Y (2002). Role of histone H3 lysine 27 methylation in Polycomb-group silencing. Science.

[15] Chase A, Cross NC (2011). Aberrations of EZH2 in cancer. Clin Cancer Res.

[16] Klionsky DJ, Emr SD (2000). Autophagy as a regulated pathway of cellular degradation. Science.

[17] Kim J, Kundu M, Viollet B, Guan KL (2011). AMPK and mTOR regulate autophagy through direct phosphorylation of Ulk1. Nat Cell Biol.

[18] Jung CH, Ro SH, Cao J, Otto NM, Kim DH (2010). mTOR regulation of autophagy. FEBS Lett.

[19] Kobayashi S, Liang Q (2015). Autophagy and mitophagy in diabetic cardiomyopathy. Biochim Biophys Acta.

[20] Kubli DA, Gustafsson ÅB (2015). Unbreak my heart: targeting mitochondrial autophagy in diabetic cardiomyopathy. Antioxid Redox Signal.

[21] Lu Z, Luo RZ, Lu Y, Zhang X, Yu Q, Khare S, Kondo S, Kondo Y, Yu Y, Mills GB, Liao WS, Bast RC (2008). The tumor suppressor gene ARHI regulates autophagy and tumor dormancy in human ovarian cancer cells. J Clin Invest.

[22] Lu Z, Baquero MT, Yang H, Yang M, Reger AS, Kim C, Levine DA, Clarke CH, Liao WS, Bast RC (2014). DIRAS3 regulates the autophagosome initiation complex in dormant ovarian cancer cells. Autophagy.

[23] Zhou X, An G, Lu X (2015). Hydrogen sulfide attenuates the development of diabetic cardiomyopathy. Clin Sci (Lond).

[24] Liu L, An X, Li Z, Song Y, Li L, Zuo S, Liu N, Yang G, Wang H, Cheng X, Zhang Y, Yang X, Wang J (2016). The H19 long noncoding RNA is a novel negative regulator of cardiomyocyte hypertrophy. Cardiovasc Res.

